# 

**Published:** 1900-08

**Authors:** 


***NOTICE.—This JOURNAL and THE INTERNATIONAL JOURNAL OF SURGERY one year for $1.00 cash, to new subscribers. Out-of-town checks not accepted unless 10c. is added for cost of collection. Strictly to new subscribers.***

**This Journal and Atlanta Semi-weekly Journal one year for $1.00 cash in advance. No checks. Strictly to new subscribers.**

**This Journal and Atlanta Semi-weekly Journal one year for $1.00 cash in advance. No checks. Strictly to new subscribers.**

**This Journal and Atlanta Semi-weekly Journal one year for $1.00 cash in advance. No checks. Strictly to new subscribers.**

